# In the June 2012 issue

**DOI:** 10.1590/S1980-57642012DN06020001

**Published:** 2012

**Authors:** Ricardo Nitrini

**Affiliations:** Editor-in-Chief

In this issue we are publishing one review, four original papers, brief reviews and the abstracts of the Third Brazilian and First Latin American Symposium on Frontotemporal Lobar Degeneration.

**Araújo et al.** carried out a systematic review of the literature on the cognitive evaluation of patients with migraine. Abnormalities in attention, memory and mental process speed have been reported by several studies from headache clinics whereas cognitive abnormalities have been less frequently reported in community-based studies. The authors concluded that further studies are warranted to verify whether there is a relationship between migraine pathophysiological process and cognitive dysfunctions.

**Silva et al.** assessed cognitive dysfunction in patients with diabetes mellitus type II (DM-II) in a case-control study. The occurrence of cognitive decline in patients with DM-II has been reported by several studies and the authors concluded that simple tests may be used for early detection of cognitive abnormalities in this very frequent disease.

**Novaretti et al.** investigated whether simple tests may be used for the differential diagnosis between the cognitive impairment secondary to depression and Alzheimer's disease. The authors showed that a brief battery that is not heavily influenced by the educational level of the patients may be suitable for this sometimes very difficult differential diagnosis.

**Brito-Marques et al.** compared the performance of elderly individuals with different schooling levels in a visual reproduction test of geometric pictures. The complexity of the pictures to be reproduced and the years of schooling interfere with the reproduction. The authors concluded that clinical evaluations using reproduction of geometric pictures must take into account the schooling level of the patients.

**Oliveira et al.** evaluated the performance of two groups of patients with Alzheimer's disease from two different Brazilian regions and observed dissimilarities in their neuropsychological profile. The authors discussed the several reasons that may account for the observed discrepancy.

In the News & Perspectives section, **Brucki** presents considerations on six recent published papers that are interesting for our readers.

**First Latin American Symposium and Third Brazilian Symposium on Frontotemporal Lobar Degeneration.** For the third time we are publishing the abstracts of the Brazilian Symposium on Frontotemporal Lobar Degeneration, which from this year incorporated the Latin American Symposium on the same subject.

**Ricardo Nitrini** Editor-in-Chief

